# Addressing a Growing Crisis: Exploring Novel Insights and Solutions in Research on Nutrition and Childhood and Adolescent Obesity

**DOI:** 10.3390/children10121883

**Published:** 2023-11-30

**Authors:** Alexandra Foscolou, Odysseas Androutsos, Rena I. Kosti

**Affiliations:** 1 Laboratory of Clinical Nutrition and Dietetics (CND-Lab), Department of Nutrition & Dietetics, School of Physical Education, Sport Science and Dietetics, University of Thessaly, 42132 Trikala, Greeceoandroutsos@uth.gr (O.A.); 2 Institute of Preventive Medicine Environmental and Occupational Health Prolepsis, 15125 Athens, Greece

Given the escalating global prevalence of non-communicable diseases (NCDs), it is consequently crucial to address childhood obesity by promoting lifestyle adjustments, as exemplified in the World Health Organization’s Package of Essential Non-Communicable Disease Interventions for Primary Healthcare [1]. Several studies have provided evidence suggesting an escalation in the frequency of overweight and obese individuals, as well as a rise in mean body mass index (BMI) among children and adolescents [2,3]. This concerning trend can be mostly attributed to the consumption of energy-dense, unhealthy and high-processed foods, as well as a lack of physical activity and excessive sedentary behavior [4,5]. In addition to these factors, it is noteworthy that parental mismanagement of children’s weight is often a significant contributor to the prevalence of childhood obesity [6]. The aforementioned conditions pose a significant detriment to the well-being of children, potentially culminating in the manifestation of chronic diseases that encompass some of the most prevalent causes of mortality worldwide, namely hypertension, certain types of cancer, type 2 diabetes, metabolic syndrome and cardiovascular disease [7]. 

The crucial role of vitamin D deficiency in exacerbating inflammatory processes in adipose tissue, as well as in impaired tissue insulin sensitivity among overweight children and adolescents is underlined in the literature [8]. Moreover, despite the fact that the focus of copious research has shed light on the importance of breakfast consumption in childhood, information on the effects of breakfast composition remains scarce [9,10]. In addition, although its role is not fully elucidated, the time of adiposity onset is among the various genetic and environmental risk factors of adulthood obesity [11]. Therefore, an accurate nutritional assessment pertaining to the early detection of obesity, as well as of any nutritional deficiency across all age groups, particularly in children and adolescents, is imperative for the prevention of overweight and obese individuals [12]. However, it is important to acknowledge that obesity is a complex disease, as it arises from a multitude of factors rather than a single cause [13]. This complexity is attributed to the intricate interplay between genetic, biological, developmental, behavioral, and environmental factors.

Thus, this Special Issue contains five articles, which have incorporated different designs and methodologies to explore new insights and potential solutions for childhood obesity. These articles, we believe, will enhance our understanding of the complex relationship among diet, lifestyle, environment, genetics, and health outcomes in children. The subsequent paragraphs provide brief descriptions of these articles.

In the narrative review by Gketsios et al. (contribution 1), the influence of parental misperceptions concerning the weight status of their children during the critical developmental stages of childhood and early adolescence was examined. Specifically, they sought to investigate the effects of these misperceptions on weight control measures employed by parents, as well as the eating behaviors of their children. The electronic search resulted in 16 studies meeting the eligibility criteria set by the authors. It was revealed that parental weight misperceptions affect the weight and eating behaviors of these children, particularly in the case of overweight children. Moreover, these parents appeared to adhere to restrictive eating plans regardless of whether they had an accurate or inaccurate belief that their child was overweight or obese. Additionally, parents who underestimated their children’s weight were more likely to exert eating pressure on them.

The study by Calcaterra et al. (contribution 2) focused on the description of the modified version of the Evaluation Deficiency Questionnaire (EVIDENCEe-Q), which was specifically tailored to the needs of children living with obesity, with the foresight that further revisions to this questionnaire may enhance its ability to forecast vitamin D deficiency in children. In total, 120 children with obesity (51% boys) were recruited from the Vittore Buzzi Children’s Hospital. The EVIDENCe-Q was administered to these children to assess its suitability for this population, while the relationship between vitamin D levels and questionnaire scores was examined to inform potential adaptations of the instrument for pediatric use. They revealed that a different tool was needed to properly identify children with obesity at risk of vitamin D deficiency across different pubertal stages or sex.

The studies by Miguel-Berges et al. (contribution 3) and Kokkou et al. (contribution 4) aimed to provide more evidence regarding the determinants of obesity in children. More specifically, Miguel-Berges et al. tried to identify the primary risk factors associated with the occurrence of overweight and obesity among Spanish children, while Kokkou et al. investigated the association between weight status and breakfast composition, with a focus on macronutrients. In the study by Miguel-Berges et al., data from 1075 Spanish children aged 3 to 12 years old (49.4% boys) were used. They revealed that a robust and statistically significant positive correlation was observed among the consumption of sweetened beverages in both genders and for all ages, the hours spent in front of a screen on a weekday basis in both genders and among older children, and body mass index (BMI) z-score. In contrast, a negative association was observed among dairy product consumption in both genders and all ages, nut consumption among females aged 6–12 years, nocturnal sleep duration on weekdays among older male and female children, and their BMI z-score. Additionally, in relation to emotional well-being and self-esteem, it was observed that girls aged 6–12 years who frequently laugh and experience feelings of happiness and self-worth had lower odds of being overweight or obese. With regard to Kokkou et al.’s work, data from 1521 Greek school-aged children (45.4% boys) were used. A noteworthy positive correlation was observed between the lipid content of breakfast and the prevalence of excessive body weight in children, contrary to the breakfast protein content, independently of the children’s level of adherence to the Mediterranean diet and physical activity status.

Finally, in the retrospective cohort study of Hazart et al. (contribution 5), 950 adults with obesity, living in France, were enrolled to determine the long-term association between overweight history—with a specific focus on the timing of obesity onset, BMI, and weight gain during adulthood—and the subsequent development of metabolic syndrome, in a population of individuals with severe and morbid obesity. Based on the time of overweight onset, the individuals were divided into three groups. It was revealed that the magnitude of weight gain may be an important proxy for the assessment of metabolic risk, independent of the obesity-onset period.

In conclusion, the studies included in the Special Issue *Research on Nutrition and Childhood and Adolescent Obesity* of the journal *Children* showed that childhood obesity remains an area of public health concern that preoccupies the scientific community, and not without reason. New aspects and new factors that contribute to the emergence of this disease are continuously identified. The dietary and weight misconceptions which the parents of overweight or obese children hold and implement play a substantial role in the progression of obesity. In addition, the composition of the foods included in breakfast for these children, along with individual foods or food groups and lifestyle factors such as sleep, screentime, and overall well-being, significantly impact their weight status. Lastly, age-specific and specialized tools are required to detect nutrition-related risks. Certainly, science and research should undoubtedly remain involved in the domain of childhood obesity so as to advance the health and quality of life of future adults. Beyond that, the need for further research is warranted in terms of the contributory role of epigenetics and the gut microbiome, as well as intrauterine and intergenerational effects on the onset of obesity [14].
